# Maternal, infant, and perinatal mortality statistics and trends in Korea between 2018 and 2020

**DOI:** 10.4069/kjwhn.2022.12.23

**Published:** 2022-12-29

**Authors:** Hyunkyung Choi, Ju-Hee Nho, Nari Yi, Sanghee Park, Bobae Kang, Hyunjung Jang

**Affiliations:** 1 College of Nursing & Research Institute of Nursing Science, Kyungpook National University, Daegu, Korea; 2 College of Nursing, Research Institute of Nursing Science, Jeonbuk National University, Jeonju, Korea; 3 Vital Statistics Division, Statistics Korea, Daejeon, Korea; 4 Department of Nursing, Catholic Kkottongnae University, Cheongju, Korea

**Keywords:** Cause of death, Infant mortality, Maternal mortality, Perinatal mortality, Republic of Korea

## Abstract

**Purpose:**

This study aimed to identify maternal, infant, and perinatal mortality using the national population data of South Korea between 2018 and 2020, and to analyze mortality rates according to characteristics such as age, date of death, and cause of death in each group. This study updates the most recent study using 2009 to 2017 data.

**Methods:**

Analyses of maternal, infant, and perinatal mortality were done with data identified through the supplementary investigation system for cases of death from the Census of Population Dynamics data provided by Statistics Korea from 2018 to 2020.

**Results:**

Between 2018 and 2020, a total of 99 maternal deaths, 2,427 infant deaths, and 2,408 perinatal deaths were identified from 901,835 live births. The maternal mortality ratio was 11.3 deaths per 100,000 live births in 2018; it decreased to 9.9 in 2019 but increased again to 11.8 in 2020. The maternal mortality ratio increased steeply in women over the age of 40 years. An increasing trend in the maternal mortality ratio was found for complications related to the puerperium and hypertensive disorders. Both infant and perinatal mortality continued to decrease, from 2.8 deaths per 1,000 live births in 2018 to 2.5 in 2020 and from 2.8 in 2018 to 2.5 in 2020, respectively.

**Conclusion:**

Overall, the maternal, infant, and perinatal mortality statistics showed improvements. However, more attention should be paid to women over 40 years of age and specific causes of maternal deaths, which should be taken into account in Korea’s maternal and child health policies.

## Introduction

Maternal, infant, and perinatal death statistics provide information on the health conditions of pregnant women, mothers, and infants and offer crucial basic data for directing public policies. Each year, Statistics Korea publishes information on maternal, infant, and perinatal deaths in the cause of death statistics, which offers a general idea of mortality trends [1]. However, these reports encompass all ages, posing limitations for analysis [1]. Furthermore, there is a high chance of omitting postpartum maternal deaths from the data [1] because the hospitals where mothers give birth and where they die postpartum may differ, unlike deaths during pregnancy or labor. Therefore, it is challenging to understand maternal deaths solely from death certificates. Similarly, fetal deaths can be omitted, unlike neonatal deaths immediately after birth. Therefore, we need to understand changing trends in mortality through a specific analysis according to age, time of death, and cause of death using the maternal, infant, and perinatal death data from the cause of death supplementary investigations, instead of using the cause of death statistics.

Analyses of causes and trends of maternal deaths in South Korea (hereafter, Korea) during specific periods have been consistently published since 2004 [2,3]. The most recent study on this topic, by Lee et al. [4], which examined the maternal, infant, and perinatal death statistics and trends between 2009 and 2017, reported that the overall maternal mortality ratio substantially decreased from 13.5 per 100,000 live births in 2009 to 7.8 in 2017. An exception was the maternal mortality ratio caused by hypertensive disorders, which showed an increasing trend, whereas both infant and perinatal mortality rates showed improvement [4]. However, a recent report by Statistics Korea stated that the maternal mortality ratio was 7.8 per 100,000 live births in 2017 but increased to 11.8 in 2020, higher than the Organisation for Economic Co-operation and Development (OECD) mean of 10.9 in 2020 [5]. Hence, a more detailed analysis is required for the period from 2018 to 2020.

This study is a continuation of the study by Lee et al. [4], which analyzed population data up to 2017. We aimed to update the maternal, infant, and perinatal death statistics and understand changes in trends from 2018 to 2020, for which data recently became available. This study will provide basic data for establishing maternal and child healthcare policies in Korea, which has a low birth rate and an increasing proportion of high-risk pregnancies. The specific research purpose was to examine (1) the maternal mortality ratio by age and time of delivery; (2) the maternal mortality ratio by causes of death; (3) infant mortality by sex, survival period, and gestational age; and (4) the perinatal mortality rate by sex, survival period, and gestational age.

## Methods

Ethics statement: This study was a secondary analysis of existing data and did not require Institutional Review Board approval or informed consent.

### Study design

This was a chronological analytic study based on maternal, infant, and perinatal mortality population data.

### Data sources

The birth and death statistics produced by Statistics Korea are complete survey data. In our study, the maternal, infant, and perinatal death data from the cause of death supplementary investigations were analyzed among the Census of Population Dynamics data from the Microdata Integrated Service [6] offered by Statistics Korea between 2018 and 2020. The mortality data were collected from death certificates, child cremation reports, and supplementary investigations on fetal deaths and causes of death. Data collection was done using the following sources: When a death certificate is registered by an administrative district to the death report and population dynamics system according to the Act on Registration of Family Relations [7], it is subsequently added to Statistics Korea database. Crematorium child/infant death reports are first registered at the crematorium and then at the city/province level according to the Act on Funeral Services [8], and eventually the death report data are added to Statistics Korea database. Finally, all data on infant deaths that occurred within a year of birth, maternal deaths during pregnancy or within 6 months of childbirth, and fetal deaths at 16 weeks or later are entered into the cause of death supplementary investigation system by medical institutions, along with additional details regarding death, pregnancy, and delivery. These are then automatically reported to Statistics Korea. To comprehensively investigate maternal deaths, supplementary investigations of the cause of death conduct further analyses at the hospitals of childbirth and hospitals of death for dead mothers for whom information was recorded on their maternal death or a delivery code was present in their health insurance records within 6 months of death. Final maternal death cases are determined through reviews by an advisory committee consisting of obstetric and pediatric experts. Child and fetal deaths are also determined through cremation report data [9].

### Definition of terms

Maternal death is a death that occurs during pregnancy or within 42 days of childbirth due to pregnancy, management related to pregnancy, or a specific cause aggravated by pregnancy, without coincidence or an accidental cause, regardless of the duration or site of the pregnancy [10]. The maternal mortality ratio is the number of female deaths that occur during pregnancy or within 42 days of childbirth due to pregnancy-related causes, divided by the number of children born that year, shown as a ratio of 1:100,000 [10].

The underlying cause of death was defined as “a disease or damage that caused a series of events that directly led to death, or accidents or violence that caused a fatal injury,” according to the seventh Korean standard classification of diseases [11]. The causes of maternal, infant, and perinatal death were derived based on these underlying causes of death.

A direct obstetric death is a death caused by obstetric complications during pregnancy, labor, and the puerperal period. An indirect obstetric death refers to death caused by an underlying disease or a disease that developed during pregnancy and was aggravated by the physiological effects of pregnancy, but not directly by obstetric causes [10].

An infant death is a death within a year of birth (365 days). The infant mortality rate is the number of infant deaths within a year of birth (365 days) divided by the number of childbirths from the same year, shown as a 1:1,000 ratio [10].

A perinatal death refers to a case of fetal or neonatal death that occurs before and after childbirth; it encompasses fetal death at the 28th gestational week or later and neonatal death within seven days of birth. The perinatal mortality rate is the number of fetal deaths at the 28th gestational week or later and neonatal deaths within 7 days of birth, divided by the total number of childbirths (defined as childbirths and fetal deaths at week 28 or later from the same year), shown as a 1:1,000 ratio [9].

### Statistical methods

The complete survey data were analyzed using descriptive statistics and calculations according to formulas based on terms’ definitions.

## Results

### Maternal deaths by maternal age and time of delivery

The maternal mortality ratio decreased from 11.3 per 100,000 childbirths in 2018 to 9.9 in 2019. However, it increased to 11.8 in 2020, reflecting an increase of 1.9 (19.2%) compared to the previous year (Table 1). The age range of maternal death was between 19 and 41 years. The maternal mortality ratio in those aged 24 years or younger was 13.7 in 2018, 8.1 in 2019, and 9.5 in 2020, showing a considerable decrease. Maternal deaths in the past 3 years mainly occurred among women in their 30s, especially in the age range of 35 to 39 years. However, the maternal mortality ratio rapidly increased in those aged 40 years and older, from 7.8 in 2018 to 14.6 in 2019 and 28.9 in 2020. This suggests a considerable increase in the risk of maternal death in women with advanced maternal age (over 40 years old).

An analysis of maternal deaths by the time of delivery showed that the proportion of antepartum maternal deaths was 10.8% in 2018, 16.7% in 2019, and 6.3% in 2020. Most maternal deaths occurred postpartum. The proportion of deaths within a day of delivery in the past 3 years was 46.0% in 2018, 46.7% in 2019, and 59.4% in 2020, showing a consistently high level. The number of deaths between 15 and 42 days was one and two in 2018 and 2019, respectively; however, it showed a considerable increase to six in 2020 (Table 1).

### Maternal mortality ratio by cause of death

Among the 99 maternal deaths between 2018 and 2020, the number of direct and indirect obstetric deaths was 75 (75.8%) and 24 (24.2%), respectively. The maternal mortality ratio from direct obstetric causes showed a steady increase, from 7.0 per 100,000 childbirths in 2018 to 8.6 in 2019 and 9.5 in 2020. Among them, 31 maternal deaths were caused by complications related to the puerperium (e.g., obstetric embolism), accounting for 41.3% of direct obstetric maternal deaths in the past 3 years. The maternal mortality ratio showed an increasing trend, from 2.5 in 2018 to 3.6 in 2019 and 4.4 in 2020. Next, the number of maternal deaths caused by complications of labor and delivery (e.g., postpartum hemorrhage) was 24, accounting for 32.0% of direct obstetric maternal deaths in the past 3 years. The maternal mortality ratio caused by labor and delivery complications was 2.6 in 2020, similar to that of 2.1 in 2018. Regarding hypertensive disorders, the maternal mortality ratio was 0.9 in 2018, 0.7 in 2019, and 1.5 in 2020, showing an increase of more than two-fold in 2020 compared to the previous year (Table 2). The three most common causes of maternal death in the past 3 years were postpartum hemorrhage, obstetric embolism, and hypertensive disorders during pregnancy, delivery, and the postpartum period (Supplementary Table 1).

### Infant mortality by sex, survival period, and gestational age

The number of infant deaths was 674 in 2020, which decreased by 257 from 2018. The infant mortality rate in 2020 was 2.5 per 1,000 childbirths, which decreased by 0.3 from 2018. In 2020, the number of infant deaths was 387 (57.4%) in male and 287 (42.6%) in female infants. A comparison with 2018 showed that the mortality rate of female infants decreased from 2.5 to 2.2, and the mortality rate of male infants decreased from 3.2 to 2.8, which was still higher than that of female infants. The neonatal mortality rate within 28 days of birth was higher than the postneonatal mortality rate after 28 days of birth in all 3 years. The postneonatal mortality rate after 28 days of birth was 1.2 in all 3 years, whereas the neonatal mortality rate within 28 days of birth was 1.6, 1.5, and 1.3 in 2018, 2019, and 2020, respectively, showing a decrease.

The infant mortality rate at less than 28 weeks decreased from 368.5 in 2018 to 300.0 in 2020; at 32 to 36 gestational weeks, it decreased from 6.1 in 2018 to 4.2 in 2020. The infant mortality rate according to gestational age between 2018 and 2020 was highest at less than 28 weeks, and the magnitude of the decrease in 2020 compared to 2018 was greatest at less than 28 weeks (Table 3). The major causes of infant death between 2018 and 2020 were certain conditions originating in the perinatal period (50.6%, 51.0%, and 48.5%, for 2018, 2019, and 2020 respectively); congenital malformations, deformations, and chromosomal abnormalities (18.7%, 16.9%, and 17.1% respectively); and symptoms, signs and abnormal clinical and laboratory findings, not elsewhere classified (NEC) (17.5%, 18.0%, and 19.4%, respectively) (Supplementary Table 2).

### Perinatal mortality rate by sex, survival period, and gestational age

The perinatal mortality rate decreased from 2.8 per 1,000 in 2018 to 2.5 in 2020. The mortality rate was higher in males regardless of year. The female mortality rate slightly decreased from 2.5 in 2018 to 2.4 in 2020, and the male mortality rate decreased from 2.8 in 2018 to 2.4 in 2020. The fetal mortality rate at 28 gestational weeks or later was higher than the neonatal mortality rate within 7 days of birth. The perinatal mortality rate at 37 to 41 gestational weeks decreased from 0.8 in 2018 to 0.7 in 2020. The perinatal mortality rate at gestational week 42 or later also decreased, from 6.8 in 2018 to 3.3 in 2020. The perinatal mortality rate according to gestational age between 2018 and 2020 was the highest at less than 28 weeks, and the extent of the decrease in 2020 compared to 2018 was greatest in week 42 or later (Table 4).

## Discussion

The number of maternal deaths in Korea has decreased in the past decade [5]. Maternal deaths since 2018 have decreased as well; however, the maternal mortality ratio was 11.8 per 100,000 live births in 2020, exceeding the OECD mean of 10.9 [5]. The increase in the maternal mortality ratio despite the decrease in overall maternal deaths is likely due to the rapid decrease in childbirths in recent years [12].

Although maternal deaths in the past 3 years were high among those aged 35 to 39 years, the risk of maternal death substantially increased in women with advanced maternal age (40 years or older). This increase may need to be considered in light of the National Supporting Program for Infertile Couples [13], which has steadily expanded its targets and extent of support since its introduction in 2006. Indeed, the birth rate of Korean women aged 40 to 44 years increased from 4.1% in 2010 to 7.1% in 2020 [12], and the rate of multiple births in women aged 40 years or older was 6.0% in 2020, a 0.8% increase from the previous year [13]. As the use of assisted reproductive technology is widespread in women with advanced maternal age (40 years or older) [14], a subsequent increase in multiple births and high-risk pregnancies can also be expected [15]. Multiple births can increase the risk of developing obstetric complications such as prenatal hypertensive diseases and postpartum hemorrhage [16], which can lead to maternal death. Therefore, the management of these obstetric complications is critical, and special attention is required for women aged 40 years and over.

The Korean government’s response to counteract maternal and infant mortality is worth reviewing. The Ministry of Health and Welfare established a management system for pregnant mothers, fetuses, and infants from pregnancy to childbirth in high-risk pregnant women, which covers 15 districts across Korea, and maternal-fetal intensive care units (MFICUs) have been introduced and operated in each district since 2014 [17]. The proportion of antepartum deaths was 6.3% in 2020, reflecting a considerable improvement compared to 10.8% in 2018 and 16.7% in 2019. This is likely due to the comprehensive management of antepartum risk factors for pregnant women by the 19 MFICUs established nationwide in 2020, except for the Jeju district [17]. However, maternal deaths within 1 day of delivery accounted for 59.4% of the total maternal deaths in 2020, which indicates that there is room for improvement in postpartum complication management immediately after delivery. Specifically, the maternal death ratio caused by complications related to the puerperium, including embolism, was 4.4 in 2020, constituting a nearly two-fold increase compared to 2.2 in 2017 [4]. Maternal deaths caused by hypertensive disorders have also steadily increased. Amniotic fluid embolism mainly occurs during or immediately after delivery, and considering that high-risk pregnancies, such as those with induced labor, advanced maternal age, and preeclampsia, are risk factors for amniotic fluid embolism [18], we can presume that high-risk pregnancies are linked to high maternal death within 1 day of delivery for the last 3 years. As a gradual increase in high-risk pregnancies, including those in women with advanced maternal age, is anticipated [19], management focused on high-risk childbirth and postpartum care will be needed all the more. Further efforts are also needed to analyze maternal mortality statistics by region and to increase access to childbirth and postpartum care, especially in obstetrically underserved areas [20].

The recent increase in rates of maternal death within 15 to 42 days of delivery was found to be high. Approximately 75% of mothers in Korea use postnatal care centers (*sanhoojori-won*) for the first 2 weeks after giving birth [21], and afterward most mothers spend the rest of the puerperium at home. Thus, the time of returning home, 15 days after delivery, appears to be when efforts to prevent maternal death are most needed. To help mothers’ postpartum recovery and newborn care, health managers are provided to families after childbirth as part of the Maternal and Newborn Health Management Support Program implemented since 2006 [22]. Mothers during this time adjust to physiological postpartum changes and must be fully aware of danger signs and symptoms, such as postpartum hemorrhage and infections, so they can immediately go to the hospital when these symptoms appear [23]. However, the checkup rate at 6 weeks postpartum is reported as 94.3%, which is lower than the prenatal checkup rate of 100% [24]. This postpartum checkup rate was even lower for mothers younger than 30 years and those between 40 and 45 years, as well as for those with low household incomes [24]. Hence, healthcare providers should offer meticulous education to prepare mothers for discharge and emphasize the importance of postpartum checkups, especially for these mothers. Furthermore, the health managers sent to each home for postpartum recovery should also be educated about postpartum risk symptoms.

The number of infant deaths gradually decreased over the 3-year period of this study, and the number of male infant deaths was 387 (57.4%). The infant mortality rate was higher in male infants (2.8) than in female infants (2.2). Since male sex has been found to be associated with an increased risk of prematurity, respiratory distress syndrome, intrauterine growth restriction, and abnormalities of the sex chromosomes [25], it may be warranted to pay closer attention to male infants in this regard. As medical services became more advanced and affordable, their accessibility has also increased [26]; with the government support system for prenatal care and universal healthcare services, the infant mortality rate in Korea (2.5) was lower than the OECD mean (4.2) [27]. According to the OECD statistics, the country with the lowest infant mortality rate among the 38 member nations was Estonia (1.4), followed by Norway (1.6); Finland and Japan (1.8); Slovenia (2.2); Czech Republic (2.3); Italy, Portugal, and Sweden (2.4); and Israel and Korea (2.5) [27]. The most frequent cause was certain conditions originating in the perinatal period (48.5%), followed in descending order by symptoms, signs, and abnormal clinical and laboratory findings, NEC (19.4%) and congenital malformations, deformations, and chromosomal abnormalities (17.1%). Conditions originating in the perinatal period, as well as congenital malformations, deformations, and chromosomal abnormalities, require pre-pregnancy and prenatal health management. However, symptoms, signs, and abnormal clinical and laboratory findings, NEC are unclear causes of death that are subcategorized into sudden infant death syndrome. As such, the incidence of mortality due to unclear causes of death appears to be increasing in Korea amid the overall decrease in infant mortality rate. Therefore, more studies are required to clarify these unclassified causes and better understand the causes of infant deaths to prepare preventive measures.

Regarding the high neonatal mortality rate within 28 days of birth, this rate has steadily decreased over 3 consecutive years and is now only slightly higher than the post-neonatal mortality rate after 28 days of birth, which was maintained at 1.2. This gradual decrease in the infant mortality rate is likely due to rapid advances in medical technology and active social investments for high-risk pregnancies and children, including advanced medical systems, a skilled medical workforce, efforts to establish and expand MFICUs and management support programs, and the 2015 medical aid sponsor program for high-risk pregnancies [26].

In light of the World Health Organization Multicountry Survey [28] data, which reported high neonatal deaths within 28 days of birth in mothers aged 35 years or older, along with gradual increases in Korea’s birth rate among women aged 40 to 44 years (reaching 7.1% in 2020), the management of pregnancies in women with advanced maternal age is critical. In addition, failing to provide proper care to infants born prematurely can further contribute to infant mortality. Given that Korea’s policies regarding premature infants are centered on postnatal support, and the procedures for preventing premature birth are still lacking [29], the public healthcare system infrastructure for premature infants should be developed, and medical, financial, and psychosocial support should be offered to families with premature infants.

As for perinatal mortality, the rate decreased from 2018 to 2020, which echoes the results reported by Lee et al. [4], according to which perinatal mortality gradually improved between 2009 and 2017. The perinatal mortality rate between 2018 and 2020 decreased among infants born at 42 gestational weeks or later, which is consistent with a report that the risk of fetal death decreases as the gestational age increases [30]. This result is likely due to improvements in prenatal survival at advanced gestational age and related postnatal survival [31], and it underscores the importance of supportive policies aimed at prolonging pregnancy to reduce fetal death.

In this study, the highest mortality rate was observed at less than 28 gestational weeks, while the greatest decrease in mortality rate was found at 42 gestational weeks or later. This is likely to have resulted from efforts to reduce the mortality rate for infants at gestational ages and birthweights with a high probability of survival [32] through prenatal care involving diagnostic tests at the initial and third-trimester prenatal checkups [33]. Therefore, in order to facilitate continued reductions in the perinatal mortality rate, policy efforts such as strengthening monitoring, expanding the scope of medical information systems, and intervening in medical services are required [34].

The finding of a higher perinatal mortality rate in male infants coincides with the results of the study by Lee et al. [4]. Likewise, Woldeamanuel and Gelebo [35] reported a higher perinatal mortality rate in male infants in Ethiopia, and perinatal death or major morbidity was more noticeable in male infants in general [27]. More studies are required to understand differences in the perinatal mortality rate according to biological differences [27]. Determining the factors that are significantly associated with the perinatal mortality rate through repeated analyses and concentrated efforts to reduce perinatal mortality will be needed in the future.

In our study, the fetal mortality rate at the 28th gestational week or later was higher than the neonatal mortality rate within 7 days of birth. Preterm delivery is the major cause of infant mortality rate [31]. A study on the perinatal mortality rate between 2015 and 2018 suggested that the correlation of perinatal mortality with chronic hypertension was mostly mediated through preterm births and that preventing preterm births caused by chronic hypertension could eliminate 87% of perinatal deaths [36]. Therefore, efforts are required to diagnose and treat various underlying diseases in addition to chronic hypertension and follow up on diseases mediated by preterm birth to prevent them.

This study followed a prior analysis comparing population data on mortality between 2009 and 2017 [4] and updated the statistics on maternal death, infant death, and perinatal mortality from 2018 to 2020. We found that advanced maternal age (40 years or older) was a crucial influencing factor for maternal, infant, and perinatal deaths [29], which echoes the results of the previous study by Lee et al. [4]. Hence, comprehensive management of high-risk pregnancy-related diseases, the complications of multiple pregnancies, preterm births, stillbirths, and premature babies will be required. Active maternal and child public health policies, such as implementing maternal and newborn health management support programs, establishing MFICUs and premature baby support programs, and continuing advances in medical technology, have enabled the active management of high-risk pregnant mothers and infants in Korea. However, the management of postpartum complications is still lacking and requires intensive supervision. Furthermore, more studies are necessary to understand the biological differences according to sex, which manifested as disparities in male infant and perinatal deaths. Endeavors are also needed to prevent infant deaths through follow-up investigations and management of infant deaths with “unclear causes,” which are increasing despite the overall decrease in infant deaths.

## Figures and Tables

**Table 1.** Maternal mortality by age and time of delivery (Korea, 2018–2020)

Variable	Categories	n, ratio, or n (%)		
		2018	2019	2020
Maternal death (total)		37	30	32
Maternal mortality ratio^†^		11.3	9.9	11.8
Age-specific maternal mortality ratio^†^	≤24	13.7	8.1	9.5
	25–29	4.6	8.6	7.9
	30–34	11.2	7.6	8.4
	35–39	16.5	13.7	16.6
	≥40	7.8	14.6	28.9
Death by time of delivery	Antepartum	4 (10.8)	5 (16.7)	2 (6.3)
	Postpartum	31 (83.8)	25 (83.3)	30 (93.8)
	Unknown^‡^	2 (5.4)	0 (0)	0 (0)
Specific time of postpartum death (day)	0–1	17 (45.9)	14 (46.7)	19 (59.4)
	2–7	4 (10.8)	4 (13.3)	4 (12.5)
	8–14	4 (10.8)	4 (13.3)	1 (3.1)
	15–42	1 (2.7)	2 (6.7)	6 (18.8)
	Unknown^§^	5 (13.5)	1 (3.3)	0 (0)

† Deaths per 100,000 live births.

‡ Unknown deaths.

§ Unknown time of death.

**Table 2.** Maternal mortality ratio by causes of death (Korea, 2018–2020)

Variable	Categories	n (maternal mortality ratio^†^)			n (%)
		2018	2019	2020	2018–2020
Type of cause	Direct causes	23 (7.0)	26 (8.6)	26 (9.5)	75 (75.8)
	Indirect causes	14 (4.3)	4 (1.3)	6 (2.2)	24 (24.2)
Detailed causes of direct death	Pregnancy with abortive outcome	1 (0.3)	1 (0.3)	0 (0.0)	2 (2.7)
	Hypertensive disorders	3 (0.9)	2 (0.7)	4 (1.5)	9 (12.0)
	Other maternal disorders	1 (0.3)	0 (0)	0 (0)	1 (1.3)
	Maternal care-related problems	2 (0.6)	1 (0.3)	2 (0.7)	5 (6.7)
	Complications of labor and delivery^‡^	7 (2.1)	10 (3.3)	7 (2.6)	24 (32.0)
	Complications related to the puerperium^§^	8 (2.5)	11 (3.6)	12 (4.4)	31 (41.3)
	Other obstetric conditions	1 (0.3)	1 (0.3)	1 (0.4)	3 (4.0)

† Deaths per 100,000 live births.

‡ Including abnormalities of the intensity of labor (e.g., uterine inertia) and hemorrhage.

§ Including obstetric embolism.

**Table 3.** Infant mortality by sex, survival period, and gestational age (Korea, 2018–2020)

Variable	Categories	n or n (‰)		
		2018	2019	2020
Live births		326,822	302,676	272,337
Infant mortality (total)		931 (2.8)	822 (2.7)	674 (2.5)
Sex	Male	534 (3.2)	465 (3.0)	387 (2.8)
	Female	397 (2.5)	357 (2.4)	287 (2.2)
Survival period	Neonatal (<28 days)	533 (1.6)	467 (1.5)	345 (1.3)
	Postneonatal (≥28 days)	398 (1.2)	355 (1.2)	329 (1.2)
Gestational age	<28	339 (368.5)	340 (383.7)	240 (300.0)
(week)	28–31	98 (52.7)	63 (35.7)	65 (41.6)
	32–36	136 (6.1)	99 (4.6)	87 (4.2)
	37–41	357 (1.2)	319 (1.2)	281 (1.1)
	≥42	1 (2.3)	1 (3.0)	1 (3.3)

**Table 4.** Perinatal mortality rate by sex, survival period, and gestational age (Korea, 2018–2020)

Variable	Categories	n (‰)		
		2018	2019	2020
Perinatal mortality (total)^†^		904 (2.8)	828 (2.7)	676 (2.5)
Sex	Male	473 (2.8)	401 (2.6)	340 (2.4)
	Female	396 (2.5)	378 (2.6)	317 (2.4)
Survival period	Fetal (≥28 GW)	559 (1.7)	516 (1.7)	464 (1.7)
	Neonatal (<7 days)	345 (1.1)	312 (1.0)	212 (0.8)
Gestational age (week)	<28	168 (182.6)	168 (189.6)	111 (138.8)
	28–31	226 (110.8)	199 (102.7)	194 (112.3)
	32–36	272 (12.0)	245 (11.2)	198 (9.6)
	37–41	235 (0.8)	215 (0.8)	172 (0.7)
	≥42	3 (6.8)	1 (3.0)	1 (3.3)

GW: Gestational week.

† Deaths per 1,000 live births; including cases where the sex was unknown.

## Supplementary materials

Further details on supplementary materials are presented online (available at https://doi.org/10.4069/kjwhn.2022.12.23).

Supplementary Table 1. Maternal deaths by major causes of death (Korea, 2018–2020)

Supplementary Table 2. Number and composition ratio of deaths by cause in infants (Korea, 2018–2020)
