# Supplementary material for: Maternal, infant, and perinatal mortality statistics and trends in Korea between 2018 and 2020
Source: Korean J Women Health Nurs. 2022 Dec 29;28(4):348–57. doi: 10.4069/kjwhn.2022.12.23 (PMC9830121; doi:10.4069/kjwhn.2022.12.23)
Supplement: Supplementary Table 1. — Maternal deaths by major causes of death (Korea, 2018–2020) [file kjwhn-2022-12-23suppl1.pdf]

Supplementary Table 1. Maternal deaths by major causes of death (Korea, 2018–2020)

Major cause of death	n or n (%)			
	2018	2019	2020	3-Year composition ratio
Miscarriage	1	1	0	2 (2.0)
Hypertensive disorders	3	2	4	9 (9.1)
Other maternal disorders mainly related to pregnancy	1	0	0	1 (1.0)
Maternal disorders related to the fetus and the amniotic cavity and possible delivery problems	2	1	2	5 (5.0)
Abnormality of forces of labor	0	0	3	3 (3.0)
Other obstetric trauma	0	2	1	3 (3.0)
Postpartum hemorrhage	7	6	2	15 (15.2)
Other complications of labor and delivery	0	2	1	3 (3.0)
Obstetric embolism	6	9	10	25 (25.3)
Other complications related to the postpartum period	2	2	2	6 (6.1)
Other obstetric conditions, not elsewhere classified	15	5	7	27 (27.3)
Total	37	30	32	99 (100)
