# Supplementary material for: Maternal, infant, and perinatal mortality statistics and trends in Korea between 2018 and 2020
Source: Korean J Women Health Nurs. 2022 Dec 29;28(4):348–57. doi: 10.4069/kjwhn.2022.12.23 (PMC9830121; doi:10.4069/kjwhn.2022.12.23)
Supplement: Supplementary Table 2. — Number and composition ratio of deaths by cause in infants (Korea, 2018–2020) [file kjwhn-2022-12-23suppl2.pdf]

Supplementary Table 2. Number and composition ratio of deaths by cause in infants (Korea, 2018–2020)

Cause of death	2018		2019		2020	
	Death n (%)	MR (‰)	Death n (%)	MR (‰)	Death n (%)	MR (‰)
Certain infectious and parasitic diseases	4 (0.4)	0	9 (1.1)	0	6 (0.9)	0
Neoplasms	15 (1.6)	0	6 (0.7)	0	4 (0.6)	0
Diseases of the blood and blood-forming organs and certain disorders involving the immune mechanism	4 (0.4)	0	4 (0.5)	0	6 (0.9)	0
Endocrine, nutritional, and metabolic diseases	7 (0.8)	0	6 (0.7)	0	4 (0.6)	0
Diseases of the nervous system	22 (2.4)	0.1	9 (1.1)	0	5 (0.7)	0
Diseases of the circulatory system	15 (1.6)	0	17 (2.1)	0.1	5 (0.7)	0
Diseases of the respiratory system	9 (1.0)	0	8 (1.0)	0	7 (1.0)	0
Diseases of the digestive system	4 (0.4)	0	4 (0.5)	0	6 (0.9)	0
Diseases of the genitourinary system	1 (0.1)	0	2 (0.2)	0	2 (0.3)	0
Certain conditions originating in the perinatal period	471 (50.6)	1.4	419 (51.0)	1.4	327 (48.5)	1.2
Congenital malformations, deformations, and chromosomal abnormalities	174 (18.7)	0.5	139 (16.9)	0.5	115 (17.1)	0.4
Symptoms, signs and abnormal clinical and laboratory findings, NEC	163 (17.5)	0.5	148 (18.0))	0.5	131 (19.4)	0.5
Injury, poisoning, and certain other consequences of external causes	42 (4.5)	0.1	51 (6.2)	0.2	56 (8.3)	0.2
Total	931 (100)	2.8	822 (100)	2.7	674 (100)	2.5

MR, Mortality rate (deaths per 1,000 live births).
